# Mechatronic Device Control by Artificial Intelligence

**DOI:** 10.3390/s23135872

**Published:** 2023-06-25

**Authors:** Martin Bohušík, Vladimír Stenchlák, Miroslav Císar, Vladimír Bulej, Ivan Kuric, Tomáš Dodok, Andrej Bencel

**Affiliations:** Department of Automation and Production Systems, Faculty of Mechanical Engineering, University of Zilina, 010 26 Zilina, Slovakia; vladimir.stenchlak@fstroj.uniza.sk (V.S.); miroslav.cisar@fstroj.uniza.sk (M.C.); vladimir.bulej@fstroj.uniza.sk (V.B.); ivan.kuric@fstroj.uniza.sk (I.K.); tomas.dodok@fstroj.uniza.sk (T.D.);

**Keywords:** agile eye, prediction, artificial intelligence, neural networks

## Abstract

Nowadays, artificial intelligence is used everywhere in the world and is becoming a key factor for innovation and progress in many areas of human life. From medicine to industry to consumer electronics, its influence is ever-expanding and permeates all aspects of our modern society. This article presents the use of artificial intelligence (prediction) for the control of three motors used for effector control in a spherical parallel kinematic structure of a designed device. The kinematic model used was the “Agile eye” which can achieve high dynamics and has three degrees of freedom. A prototype of this device was designed and built, on which experiments were carried out in the framework of motor control. As the prototype was created through the means of the available equipment (3D printing and lathe), the clearances of the kinematic mechanism were made and then calibrated through prediction. The paper also presents a method for motor control calibration. On the one hand, using AI is an efficient way to achieve higher precision in positioning the optical axis of the effector. On the other hand, such calibration would be rendered unnecessary if the clearances and inaccuracies in the mechanism could be eliminated mechanically. The device was designed with imperfections such as clearances in mind so the effectiveness of the calibration could be tested and evaluated. The resulting control of the achieved movements of the axis of the device (effector) took place when obtaining the exact location of the tracked point. There are several methods for controlling the motors of mechatronic devices (e.g., Matlab-Simscape). This paper presents an experiment performed to verify the possibility of controlling the kinematic mechanism through neural networks and eliminating inaccuracies caused by imprecisely produced mechanical parts.

## 1. Introduction

Every day, buildings or people are monitored by video surveillance systems. Currently used security cameras placed on buildings are sometimes able to detect people or animals in the image through machine vision and even rotate the camera to keep such objects in view according to the movement of the objects being monitored. Achieving the desired camera movement is ensured by the motors implemented in the design of the camera mount. In some applications intrusion detection, a critical component of network security, may monitor network activities as well as detect intrusions/attacks. The control of the motors is provided by the camera control unit, which is programmed according to the specific kinematic structure of the mechanism. According to the kinematic model and the type of motors used in construction, it is possible to find the dependencies and equations for the control, according to which the specific movement of the individual motors can be recalculated for every possible movement [1,2].

Nowadays, various applications of artificial intelligence are becoming more and more prominent. In particular, applications of machine vision (categorizing objects in a captured image), prediction (predicting values that may occur), and voice control, based on recognition of spoken commands, are frequently applied in industry. The stated applications become an integral part of Industry 4.0 and are suited to be deployed in various industrial applications such as machine manufacturing, packaging, the automotive and food industries, and many others. The primary significance of integrating machine vision with predictive capabilities lies in the mitigation of errors caused by human interventions, while harnessing a sufficient level of accuracy and stability that surpasses the limits attainable through human effort alone. The implementation of artificial intelligence results in a decrease in error rates and undesirable production downtimes. Additionally, it enhances the efficiency of production, facilitates traceability, and increases workplace safety [3].

This paper focuses on the implementation of predictions in controlling the motors of a still-quite-rare kinematic structure, “Agile eye” (see Section 2.1), through prediction, using artificial neural network models. In this research, artificial neural network models address the task of approximating a continuous function that describes the kinematics of the mechanism.

Mechatronic devices (their actuators–motors) are often controlled according to complex kinematic equations resulting from Euler angles/Tait-Bryan angles, Matlab, based on implemented sensors, etc. Our objective was to investigate the feasibility of bypassing the utilization of these equations and substituting them with neural networks (prediction) as an alternative approach. In addition to the device performing the required movements after prediction, a calibration was created, after which the device performed more accurate movements—Section 3.4.

The device design incorporated existing manufacturing technologies at the university, to ensure the feasibility of its production. The main means of production were a Prusa i3 MK3S+ 3D printer (Prusa Research, Prague 7, Czech Republic, the device was purchased through their eshop), an EMCO Concept Turn 55 lathe (EMCO GmbH, Salzburger Str. 80, Hallein-Taxach, the device was purchased through their dealer), and a Z7016 Vario bench drill (CORMAK, Brzeska 120, Siedlce, the device was purchased through their eshop).

In previous AI projects, we have predominantly used Python as the programming language. To create the control program in this application, Python 3.8.15 and available software libraries were used. Some of the libraries that can be used for prediction include scikit-learn (MLPRegressor), pandas, matplotlib, and numpy.

Scikit-learn is an open-source software library that offers several features in the field of machine learning. The most used functions include classification, regression, clustering, dimensionality reduction, model selection, etc. [4].

Pandas is an open-source data analysis and manipulation tool that is quick, powerful, flexible, and simple to use. This library contains several features suitable for machine learning projects. We used the “reading and writing data” function, which gave us access to a .csv file that contained the coordinates of the points where we wanted the optical axis of the effector to move. The neural network training was carried out by reading values from the .csv file where five columns were created, where there were two inputs (X,Y) and three outputs (M1, M2, M3). More details about the neural network training are summarized in Section 2.2 [5].

Matplotlib was used to plot the training progress on a graph. NumPy is the foundational Python library for scientific computing. It is a Python library that includes a multidimensional array object, various derived objects (such as masked arrays and matrices), and a variety of routines for performing fast array operations such as mathematical, logical, shape manipulation, sorting, selecting, I/O, discrete Fourier transforms, basic linear algebra, basic statistical operations, random simulation, and much more. This library, due to its wide possibilities of use, was used in all the created scripts that were related to the control of the created device [6].

The paper’s contribution lies in the development of a methodology to control a mechatronic device through prediction while creating a calibration to obtain more accurate effector positions. The paper also provides an overview of the different effector motion options of described device, including the Fourier transform.

**Figure 1 sensors-23-05872-f001:**
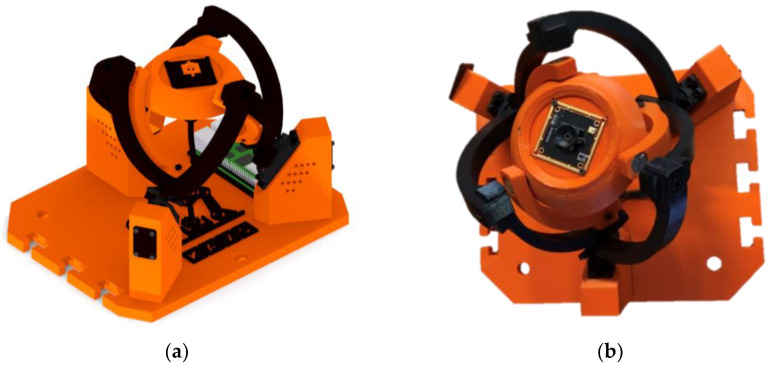
Agile Eye design: (**a**) CAD model; (**b**) physical construction of the device.

In Figure 1a, the design of the device used for the experimental part of this project is shown. Figure 1b shows the prototype device constructed. The parts shown in black and orange were printed from PETG material. The joint pins used in the rotating kinematic pairs were turned on a lathe. On the right side of the device base, “T-cuts” were created to allow for attaching another device for presentation purposes while maintaining the distance between the axes of the effectors. Such a connection of two devices enables the device to be repurposed for further research in the field of stereovision. In practice, the outputs can be used to measure the distance of objects from the device or the dimensions of tracked objects, detect the speed of moving objects etc.

The next part of the research will consist of object recognition of the workspace of the 3D-measuring device through machine vision. The device will be able to detect the object to be measured, the measuring touch probes and the order of placement of the measuring probes in the tool stack. Another project will focus on the navigation of a mobile robot by tracking a specific person via machine vision or by voice commands of a specific person.

## 2. Materials and Methods

Currently, it is possible to find several published research studies in the field of the above-mentioned motor control methods. One of them is, for example, the Model-Based Design of Induction Motor Control System in MATLAB, where the author’s team utilized a mathematic model of an induction motor based on Kirchhoff’s second law with the consideration of Maxwell’s equation [7].

Despite many publications in the field of motor control using predictions, our solution offers to some extent the possibility of eliminating overall inaccuracies and backlash in the mechanism through calibration. The publications currently available in the field of motor control utilizing neural networks, and which are relevant to the problem addressed in this paper, can be categorized as follows:Kinematic Control of Redundant Manipulators Using Neural Networks [8].From neuron to behavior: dynamic equation-based prediction of biological processes in motor control [9].Neural Network-Based Motion Control of Underactuated Wheeled Inverted Pendulum Models [10].

At first, the conditions were specified: a device with a parallel kinematic structure was created. In the control system, standard kinematic relations were replaced by prediction using neural networks. To fulfill such requirements, current materials, and methods in the field of parallel kinematics of mechatronic devices, artificial intelligence and processing of measured values were researched. The selected kinematic mechanism consisted mostly of parts created by FDM. The selected manufacturing methods resulted in mechanical errors such as inaccuracies of parts, backlash, and flexing. These errors were partially eliminated by calibration as shown in Section 3.4.1.

Although the device could have been controlled through the use of rotation matrices or according to a model created in Matlab (Section 3.2.2), we sought to replace this with inverse kinematics followed by prediction. Controlling the motors through prediction was not expected to be more accurate than controlling the mechanism through the Simscape model (Matlab). An important aspect of this study involved the incorporation of calibration, whereby the neural network is trained based on the actual detected movements. Such a training process enables the effector to achieve greater positional accuracy by compensating for any clearances in the pins or motors.

The procedure for creating the experimental part was as follows:Designing and building a mechatronic device with a parallel kinematic structure.Obtaining enough values to control the motors using inverse kinematics (CREO).Building a structure where a millimeter graph paper was placed on which the motions of the device could be studied.Writing the necessary programs.Processing the results.

The speed of movement for all types of movements on a circle or on a line between two points can be calculated as follows—all created paths after the movement are executed by the device in 36 s, so, as long as we know the length of the path, we can calculate the exact speed of the effector’s movement. The control can also be carried out the other way around—we choose the desired speed, and the number of points along which the optical axis of the effector will move is adjusted according to linear/circular interpolation. The predicted values of the servomotor rotation can be included as variable parameters. In our experiment, these were varied continuously according to the required movements of the optical axis of the device to a specific position. The measured parameter was the movement of the intersection of the optical axis of the effector on the investigated plane. The values obtained in this manner were processed afterward and are included in the results of this paper.

### 2.1. Agile Eye

**Figure 2 sensors-23-05872-f002:**
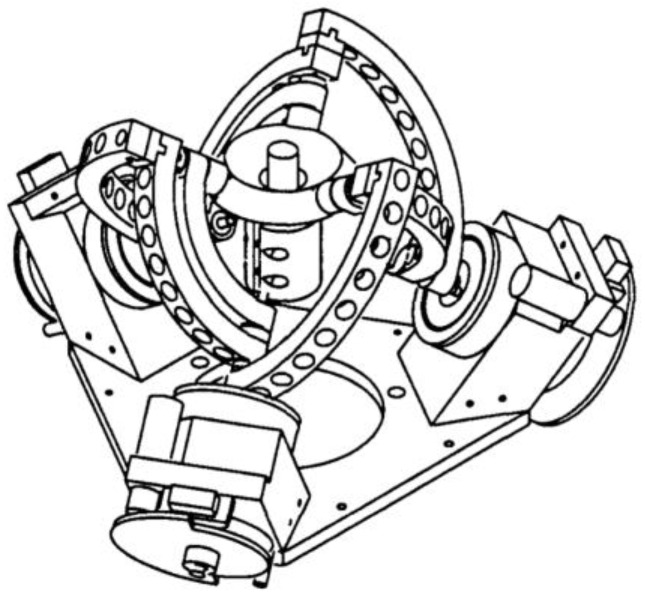
Design of Agile Eye mechatronic device [11].

The kinematic model of the agile eye is a high-performance three-degree-of-freedom camera-orienting device that was designed at Université Laval in 2002. The original model achieved a relatively high positioning speed. The device can be used in various applications. For example, it can be used for positioning effectors such as a camera for real-time tracking of objects, or a laser or mirror to project light beams onto a certain surface. The design of this device is shown in Figure 2 [11].

The studies referenced in [11] point out that to achieve the combination of two camera images into a stereopair, it is necessary for both cameras to contain three degrees of freedom. Given that this is an implementation of AI algorithms into a mechatronic device, preparing the implementation of other AI methods, such as machine vision and voice recognition (an Arducam IMX179AF—Arducam, Nanjing, China, the device was purchased through their dealer), has been taken into account. Such mechanisms are used in the heads of humanoid robots, where through the stereo vision device, the robot is able to track the scene around it and at the same time it can detect the distance of the detected objects from the sensing device (camera). The preparation of stereovision can lead to the creation of new research in the investigated field.

The control of this mechanism consisted in expressing the Tait-Bryan equations on all links of the mechanism. The whole study regarding the construction of this mechanism, the kinematic analysis, and its control is written in a study detailed in reference [12]. The mentioned study proves that the Agile Eye’s workspace is limitless and is only flawed by six singularity curves (as opposed to surfaces). These curves were also found to match the mobile platform’s self-motions [12].

### 2.2. Neural Network Model

The model that was used to calculate the positions of all 3 servomotors is a multilayer perceptron, which consists of 1 input layer, 1 hidden layer, and 1 output layer. The input layer is composed of 2 artificial neurons, where each neuron belongs to the desired position to which the mechanism is to be directed. The hidden layer contains 500 artificial neurons. The output layer contains 3 artificial neurons belonging to the 3 servo motors of the servo mechanism [13,14].

This model spreads the input signal forward through the hidden layer, and it uses some simple calculations such as weighted input and transferring this weighted input via the activation function. Those activation functions can be specific for each layer of the neural network and can often affect the final performance of the network after the training or can affect the overall training time. The most common activation functions are a hyperbolic tangent, logical sigmoid, linear function, or rectified linear unit activation function. In addition, some of the activation can not prevent problems often known as vanishing or exploding gradients problems, when the network is unable to train on the given data set.

**Figure 3 sensors-23-05872-f003:**
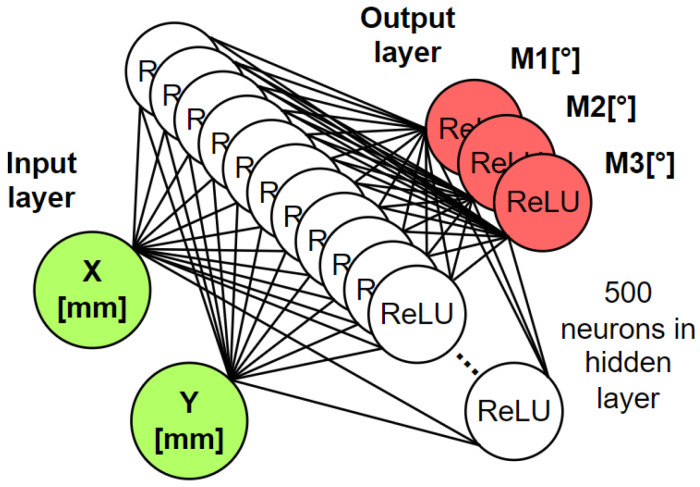
An artificial neural network architecture that approximates the kinematic model of a parallel mechanism.

All layers that were created have the ReLU—Rectified Linear Unit activation function. Testing other activation functions was not the object of this research. This activation function, compared to activation functions such as a logical sigmoid or hyperbolic tangent, better avoids the problem of vanishing gradients in the learning process. For the ReLU activation function f, the Relations (1) and (2) hold [15].
(1)$$f\left(x\right)=x^{+}=\max\left(0,x\right)=\left\{\begin{array}{c} x\;\mathrm{if}\;x>0,\\ 0\;\mathrm{otherwise}. \end{array}\right.$$
(2)$$f^{\prime }\left(x\right)=\left\{\begin{array}{c} 1\;\mathrm{if}\;x>0,\\ 0\;\mathrm{if}\;x<0. \end{array}\right.$$
where *x* represents the value of the input that enters the artificial neuron. If the input is less than or equal to 0 the output of this function is also equal to zero. If the input is greater than 0, the output of the function is equal to the value of the input. The architecture of the neural network model is shown in Figure 3.

Every training process of the neural network starts with randomizing the bias and weight parameters randomly or by some specific rule. In the terms of the initialization of the weight parameters a weight normalization rule was used. This normalization was specific for the layers with the ReLU activation function. If we were to use a logical sigmoid or hyperbolic tangent in this research a Xavier initialization method or Normalized Xavier initialization method could be used. Because of these random-based initialization methods, each training could achieve different results in the terms of the overall performance of the network.

The training continues with slowly changing the initialized weight and bias parameter values according to the error function. The final goal is to reach a global minimum in the error space for a specific situation. The most often used error functions (also referred to as cost functions) are mean squared error MSE or root mean squared error function RMSE. The standard training methods such as gradient descent or Levenberg-Marquardt optimalization algorithms set up all the parameters so the derivative of this function according to weight and bias parameters is equal to zero. In this research a dataset was created for learning with a teacher.

**Figure 4 sensors-23-05872-f004:**
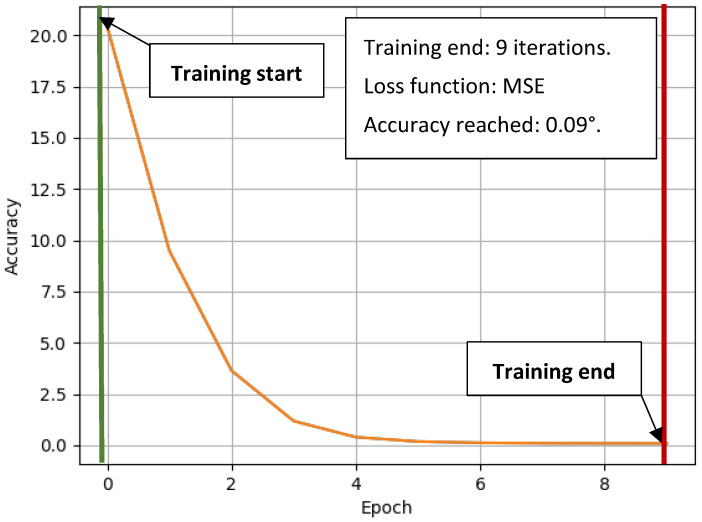
Training progress of a neural network by the Adam training method.

The X and Y variables are the values of the X and Y axis coordinates to which the target point of the effector mechanism is to be directed. M1, M2, and M3 are the rotation angles of the servomotors of the device. The training data had the format expected outputs—attributed outputs. Therefore, this is learning with a teacher. The Adam—Adaptive Momentum learning technique was used to train the model. The training was completed after 9 iterations, and the resulting accuracy (or MSE error function value) was 0.09 (shown in Figure 4.). The number of samples of the training dataset was 3600. In this project, the neural network was trained using the Scikit-learn library, a widely utilized tool [16,17].

**Figure 5 sensors-23-05872-f005:**
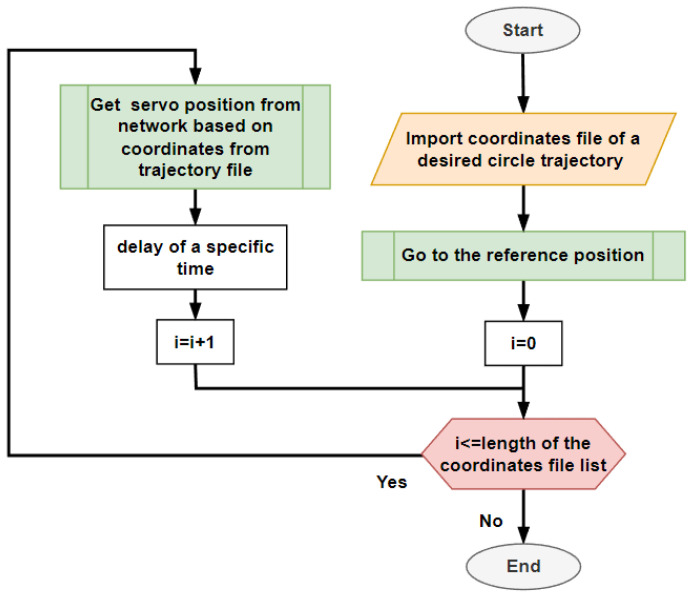
Using neural network as a controller for the servo motors.

After the training was finished the neural network could be used as a controller for the designed mechanism. A script was created for controlling the servomotors. It was necessary to provide a .csv data set with the required points of the path and after that it could be used as described in the following flowchart in Figure 5 below.

The script loads the data file containing the required positions to which the mechanism should position. After that, the neural network calls for a loop and calculates the desired servomotor positions. To give time for the mechanism to position the effector of the device, a time delay is added to the microcomputer. This time affects the final speed of the positioning the servomotors. For the purposes of this experiment a time of 100 ms was used. After the whole data list is read, the script is ended and can be called to repeat the given task again.

## 3. Results

This section contains the practical outputs of the project, in terms of device design and construction, measured data collection, and its evaluation. For purposes of this paper, the outputs of the experiment performed by Alibakhshi and Mohammadi Daniali, focused on the forward kinematics of the manipulator and usage of neural networks, including the methods proposed by Gosselin and Gagne, were analyzed. The goal of the respective experiment and the task of neural networks was “to estimate the distance between gripper and singularity or obstacle in Euler coordinate” [18,19].

### 3.1. Agile Eye Design

The basic template for developing the design was the study referred to in Section 2.1. This avoided the creation of potential errors such as the creation of two fixed circular arcs and so on.

The design used in this paper was based on the above-mentioned design and improvements and modifications such as:Servomotor cooling;Effector designed so that it can be used to mount both camera and laser;Created T-slots for precise attachment of the second device, in the case of stereo imaging testing;Mounting holes that allow mounting of the device to the wall.

Holes were not created in the individual arms in terms of reducing the weight of the device, due to the creation of the majority of the device by 3D printing (FDM). These parts encompassed a smaller fill, so the weight would be similar to that of the holes created with the full fill of the objects

#### 3.1.1. Main Components of Construction

**Figure 6 sensors-23-05872-f006:**
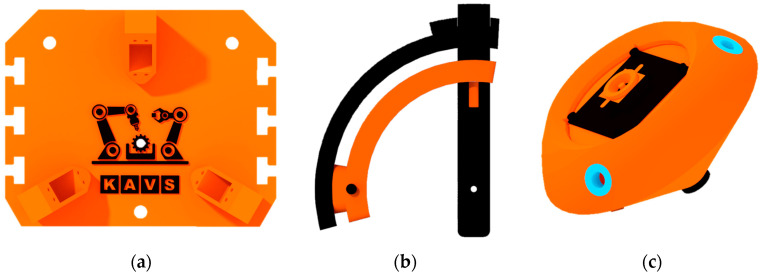
Individual parts of the Agile Eye: (**a**) base; (**b**) arm assembly; (**c**) effector.

The mechanical construction was designed to be manufactured mainly by FDM 3D printing. The simplicity of the design is important as it makes the future pairing of two mechanisms side by side (stereo vision) easier. The structure contains standard fasteners (bolts, nuts) and flanged bushings (blue color—Figure 6c).

All parts were made using a Prusa MK3S printer in five production runs:1× base (Figure 6a)—1 production cycle;3× arm assembly (Figure 6b)—3 production cycles;1× effector (Figure 6c)—1 production cycle;

During the print of the base two material changes were made for aesthetic reasons in order to increase the contrast of the departmental logo.

#### 3.1.2. Electric Components

The agile eye is a mechatronic system in which mechanics, electronics, and IT are combined. The electronic components used in this design are:3× MG996R Servo (RPishop.cz, Roudné, Czech Republic, the device was purchased through their eshop)—servo motors which are used to create torque on the device hoop and then move the effector;1× RaspberryPi 4B (RPishop.cz, Roudné, Czech Republic, the device was purchased through their eshop)—the control unit in which the scripts were created to control this mechanism, including training the neural networks;1× Servo Driver—a device that allows control of up to 16 servo motors.

The device can be used for different applications, according to which appropriate type of effector is selected. Either an Arducam 8MP IMX179AF camera or a laser, with a body diameter of 10 mm, can be attached to the proposed effector.

### 3.2. Kinematics

In many applications, mechanical systems that enable a rigid body to move relative to a fixed basis are crucial. A rigid body in space has the ability to move in both translation and rotation. The degrees of freedom (DoF) refer to the motions. A rigid body can only have a maximum of six degrees of freedom (DoFs) in space, or three translations and three rotations about the x, y, and z axes in Cartesian coordinates. A system can be referred to as a robot as soon as it is able to operate the end-effector’s multiple DoFs using a mechanical system [20].

**Figure 7 sensors-23-05872-f007:**
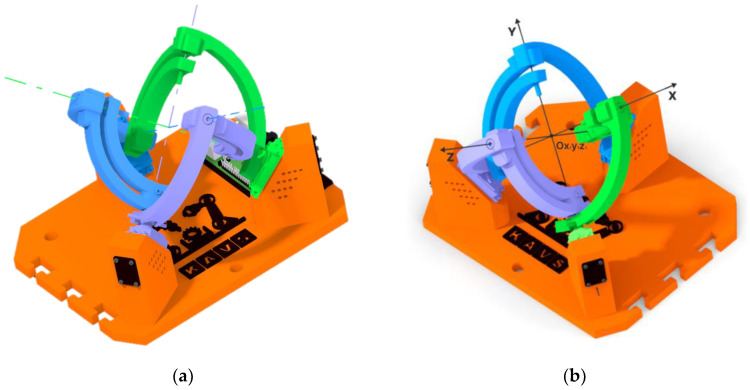
Reference configuration of the Agile Eye mechanism: (**a**) Motor axes; (**b**) Coordinate system of the device.

The Agile eye device has a parallel kinematic structure that utilizes three motors to rotate the effector with 3DoF. To achieve control of this device through prediction, it was important to find out the rotation values of the motors at different effector orientations. The underlying coordinate system, denoted Ox_1_y_1_z_1_, is symbolized by the colored axes of the individual motors which are shown in Figure 7a. The aforementioned coordinate system and its orientation can be seen in Figure 7b (the location of its axes, located in the axes of the motors).

Tait-Bryan angles were used to obtain the resulting rotation matrix while defining the orientation of the Ox_2_y_2_z_2_ coordinate system on the effector. The rotation method of this coordinate system is in the order Z_1_Y_2_X_3_, where indexes 1,2,3 symbolize the angles ψ, θ, ϕ, that were used for the rotation about each axis [21,22].

**Figure 8 sensors-23-05872-f008:**
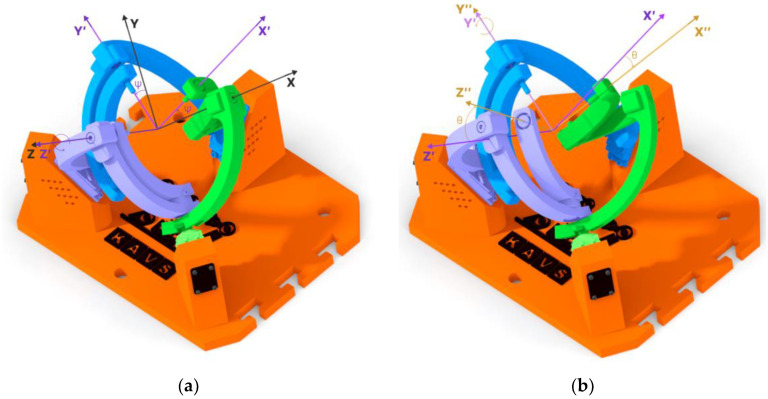
Rotation relative to the Z and Y axes: (**a**) Z-axis rotation = 20 deg; (**b**) Y-axis rotation = 20 deg.

**Figure 9 sensors-23-05872-f009:**
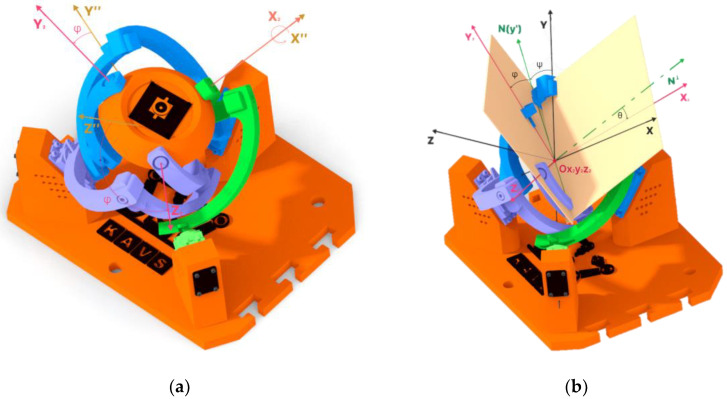
Using Tait-Bryan angles: (**a**) Rotation of the X = 40 deg axis; (**b**) Representation of the rotation angles of each axis between Ox_1_y_1_z_1_ and Ox_2_y_2_z_2_.

The principle of using Tait-Bryan angles Z_1_Y_2_X_3_, is represented in the following figures (Figure 8 and Figure 9). Figure 8a shows the rotation of the coordinate system Ox′_1_y′_1_z′_1_ (purple color) concerning the Z-axis. The angle used is defined by the symbol ψ. The second rotation (Figure 8b) occurred with respect to the Y-axis, producing the coordinate system Ox″_1_y″_1_z″_1_ (yellow color), where the angle is defined by the symbol θ.

The last (third-read) coordinate system, Ox‴_1_y‴_1_z‴_1_ (hereafter referred to as Ox_2_y_2_z_2_), was created by the rotation of the X-axis, as symbolized in Figure 9a. The angle of rotation is defined by the symbol ϕ. Specifically, the rotation angles of the coordinate system Ox_2_y_2_z_2_ are as follows:ψ = 20 deg;θ = 20 deg;ϕ = 40 deg.

These angles can also be read from Figure 9b, where two planes have been created that intersect each other. The first plane was created on the coordinate system Ox_1_y_1_z_1_, on the X and Y axes. The second plane was created on the coordinate system Ox_2_y_2_z_2_ on the Y and Z axes. At the location where these planes intersect, the N(y′) axis was created, with the N axis perpendicular to it (green colors). To define the angles between these two coordinate systems, Tait-Bryan angles were used [23].

To represent the resulting coordinate systems, three 3 × 3 rotation matrices were created. The rotation matrices created and the method of calculating the resulting rotation matrix are defined by Relation (3).
(3)$$R_{Z}\left(\psi \right)=\left[\begin{array}{ccc} cos\psi & -sin\psi & 0\\ sin\psi & cos\psi & 0\\ 0 & 0 & 1 \end{array}\right],\;R_{Y}\left(\theta \right)=\left[\begin{array}{ccc} cos\theta & 0 & sin\theta \\ 0 & 1 & 0\\ -sin\theta & 0 & cos\theta \end{array}\right],\;R_{X}\left(\varphi \right)\left[\begin{array}{ccc} 1 & 0 & 0\\ 0 & cos\varphi & -sin\varphi \\ 0 & sin\varphi & cos\varphi \end{array}\right]$$

Multiplying the rotation matrices R_Z_, R_Y_, and R_X_, we obtain the resulting rotation matrix (4) R_ZYX_, which expresses the orientation of the coordinate system Ox_2_y_2_z_2_ with respect to Ox_1_y_1_z_1_ [24].


(4)
RZYXBA=RZ(ψ)RY(θ)RX(φ)=cosψ⁡cosθcosψ⁡sinθ⁡sinφ−cosφ⁡sinψsinψ⁡sinφ+cosψ⁡cosφ⁡sinθcosθ⁡sinψcosψ⁡cosφ+sinψ⁡sinθ⁡sinφcosφ⁡sinψ⁡sinθ−cosψ⁡sinφ−sinθcosθ⁡sinφcosθ⁡cosφ


#### 3.2.1. Creo Parametric

In order to use prediction in the training of neural networks for the control of the constructed device, it is necessary to create a dataset of values that will be used for the training of the neural network. A model of the device, including the creation of the constraints, was created in Creo Parametric (CREO) to realistically represent the movements it can perform. CREO offers a “Mechanism analysis” option where the inverse kinematics can be used to obtain specific motor rotation values for each effector rotation.

**Figure 10 sensors-23-05872-f010:**
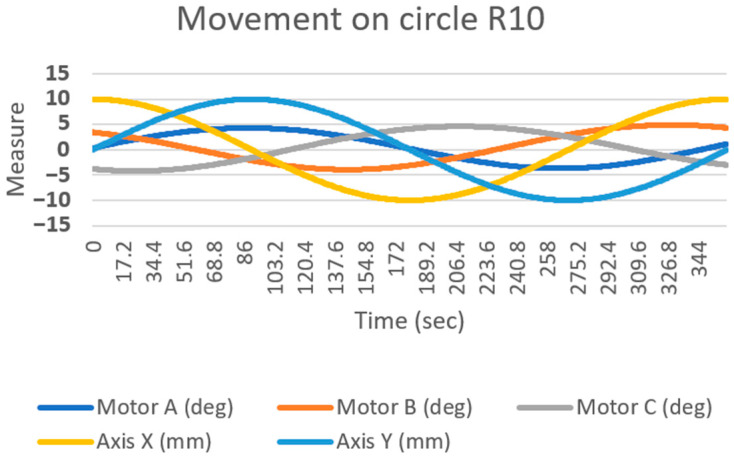
Graph of processed values of inverse kinematics, when the axis of the effector moves in the shape of a circle of radius R10.

To obtain the values of the rotation of the motors (deg), several effector movements were generated, with five values recorded every 0.1 s—the rotation of the three motors (A, B, C) and the exact location of the effector axis on the surface under study (X and Y axis). A graph of the obtained values (shown in Figure 10), was created from the values generated while the effector was performing a movement in the shape of a circle with a radius of 10 mm. The motion on the circle was generated through the goniometric function of the sine and cosine of the angle.

#### 3.2.2. Simscape Multibody Model

There are several options for controlling this mechatronic device. One of them is the use of Simulink, which can be used within Matlab. The “Simscape multibody model” extension was used to create the simulation model. By using Simulink, a direct kinematic solution for the designed mechatronic device occurs.

**Figure 11 sensors-23-05872-f011:**
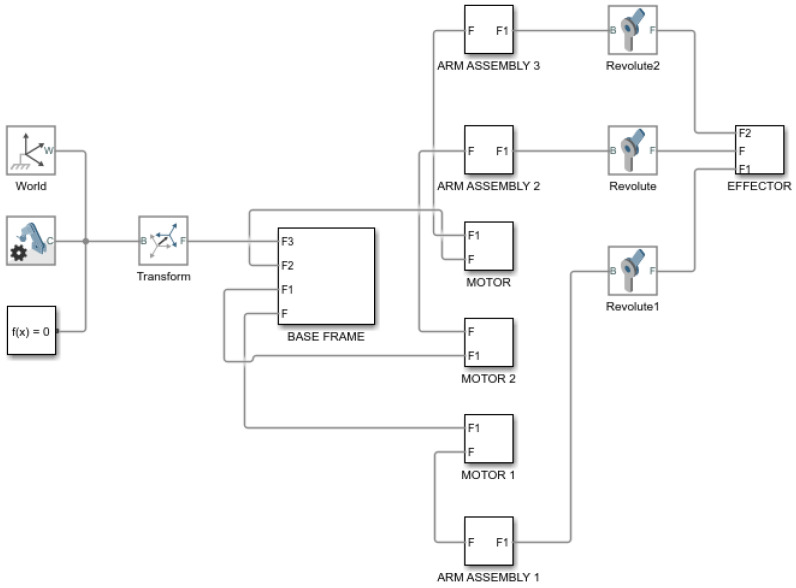
Simulation model of the Agile Eye device created in Simscape toolbox.

After exporting the data and subsequent editing, a simulation model of the device (Figure 11), was created to represent the control of this mechatronic device. The simulation model consisted of 6 functional blocks.

The simulation model created in Matlab can also be used to verify mathematical equations related to kinematics, simulate motions and evaluate numerical calculations for inverse kinematics [24].

### 3.3. Collection of Data from Created Device Movements

**Figure 12 sensors-23-05872-f012:**
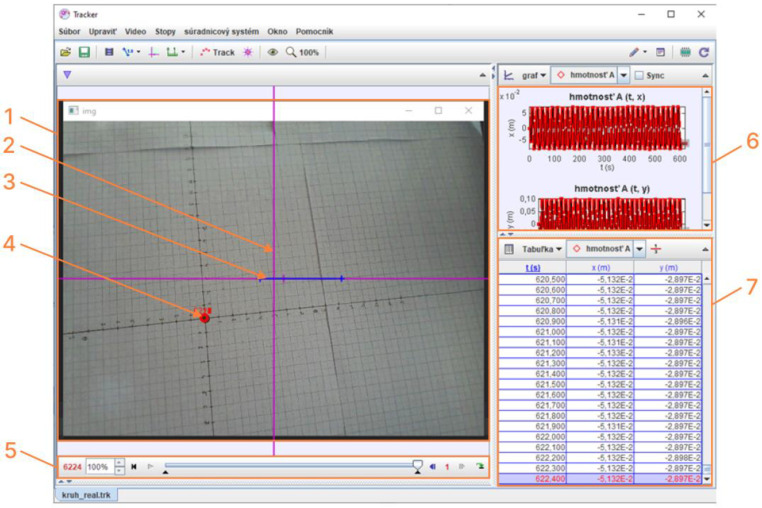
Analysis of video in the Tracker software. The video section (1) contains a coordinate system (2) relative to the camera frame, the calibration stick (3) defining the real scale of objects in the projection plane, and a point (4), the position of which is tracked in coordinates relative to the camera frame. The position of the target point is recorded using the Autotrack function in each of 6224 frames of the video, as shown in (5), and the position is recorded in table (6) and charts for individual axes (7).

After the device control was created, a program was used by which the device performed a circle through the effector axis with a radius of 20 mm (R20). During the movement of the effector, the recording of the captured image was started (Figure 12). The recorded image was analyzed in the Tracker software (Section 3.3.1). Every 0.1 s the coordinates of the tracked point (X,Y) were recorded. The coordinates obtained from the scan point tracking were processed into graphs and then evaluated.

#### 3.3.1. Tracker

Tracker is a free video analysis and modeling tool built on the Open Source Physics (OSP) Java framework. It is designed to be used in physics education. This software allows the user to manually or automatically track the position of an object in individual frames of video and thus calculate its velocity and acceleration [25].

The actual inspection of the video recorded by the camera on the agile eye itself is shown in Figure 12. 

In cases when the target mark was too blurry to be recognized by the auto-tracking algorithm, points were selected manually or omitted depending on the level of blurriness. The described setup allowed the measurement of the positioning accuracy of the agile eye mechanism without complicated and labor-intensive processes [26].

#### 3.3.2. Calculation of Achieved Points

The processed image data was processed through MATLAB. Since the training data was taken on a plane that was 74 mm away from the plane in which the camera was located, while experimenting, we had the plane from which we were detecting the eye rotation 255 mm away. Therefore, it was necessary to convert the acquired data into *b* values that were projected on a plane that was 74 mm away from the camera plane. For the angle *α*, relation 5 holds.
(5)$$\cos\alpha =\frac{255}{\alpha }$$

The *a* value was the distance projected on a plane 255 mm away from the plane in which the camera was located. In order to compare the desired position with the actual position, we needed to calculate the distance *b*. To calculate the distance *b*, Equation (6) was used.
(6)$$b=74\frac{1}{\cos\alpha }=74\frac{1}{\frac{255}{\alpha }}=\frac{74\alpha }{255}$$

**Figure 13 sensors-23-05872-f013:**
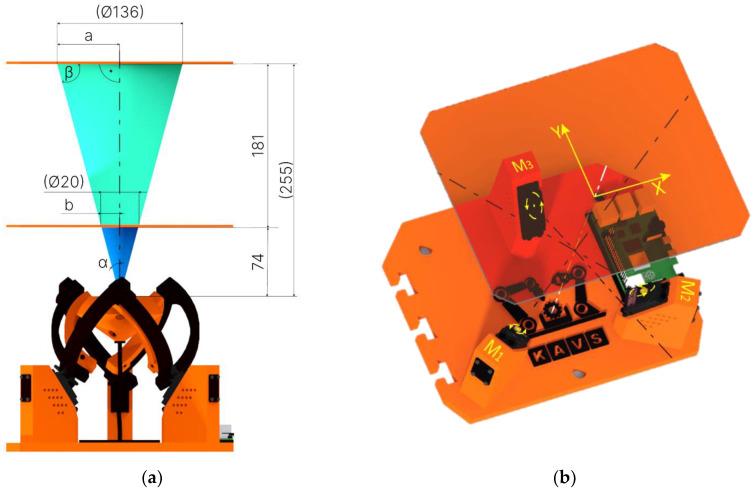
Representation of focal length and working planes: (**a**) Converting the values obtained to the distances with respect to which the mechanism was positioned (**b**) Rotating the motors M1, M2, M3, and positioning the camera to the X and Y coordinates.

A principled representation of the conversion is shown in Figure 13a.

### 3.4. Using Prediction for Motor Control

The neural network that was used in this research solved the approximation of the unknown function f. Its shape is shown in Equation (7) [27].
(7)$$y\left(M_{1},M_{2},M_{3}\right)=f(X,Y)$$

This function described the dependencies between the desired camera position given by the X and Y coordinates and the three rotations of the servomotors M1, M2, and M3 as can be seen in Figure 13b. These dependencies are described by a kinematic model. The neural network model used was able to abstract the individual dependencies from the data that was obtained from the CREO Parametric environment. In this way, it was possible to bypass the kinematic model, which is described in more detail in Section 3.2.

#### 3.4.1. Processing the Results by Moving along the Circle R20

In a two-dimensional space, there were points with X and Y coordinates on which we performed the convex wrapping. This envelope was the intersection of all convex sets containing points with X and Y coordinates. To describe the accuracy and behavior of the model that controlled the mechanism’s motors, it was necessary to calculate the radius and coordinates of the center of the smallest inscribed circle and the radius and coordinates of the largest inscribed circle. Based on these two circles, it was also possible to calculate the center circle, which was important in the calibration of the neural network model. For a center circle whose diameter is *d_mean_* and whose center coordinates are the same as those of the *d_max_* and *d_min_* circles, the following prescription can be used.
(8)$$d_{mean}=d_{min}+\frac{d_{max}-d_{min}}{2}$$

Circles with diameter *d_min_* and *d_max_* have the same center coordinates.

The behavior of the mechanism when positioning the camera along a circular path can also be described by the geometric tolerances prescribed by STN EN ISO 1101:2006. These geometric tolerances are divided into 4 categories according to the standard: geometric tolerances of shape, position, orientation, and throw [28,29].

The circularity tolerance, which belongs to the group of shape tolerances, was used to describe the observed trajectory. The tolerance zone for this tolerance tends to be bounded by two concentric circles. Their radial distance is the prescribed value of this tolerance. This parameter was used in the research to describe the positioning accuracy of the parallel kinematic structure.

**Figure 14 sensors-23-05872-f014:**
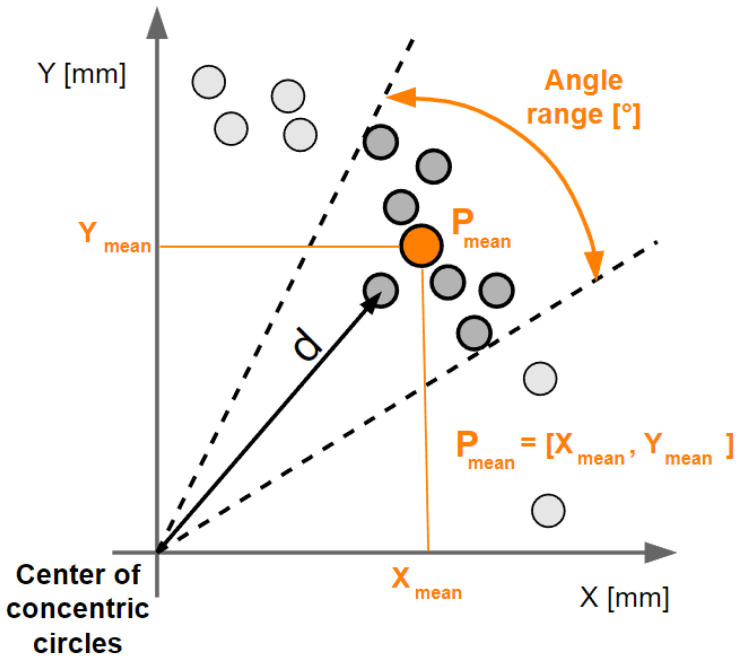
Illustration of the processing of the average trajectory from 30 repetitions of the circular motion.

For calibration purposes, the measured data also had to be processed. The equipment used performed 30 repetitions for each diameter. From these 30 repetitions, an average trajectory was created. This path was made up of 360 points, each point belonging to a certain angular range. In our case, this was a 1° angular span, as can be seen in Figure 14.

To calculate the average trajectory formed by the *P_mean_* points, it was necessary to calculate the distance of the points from the center of the inscribed and circumscribed circle. The relation for calculating the distance d, which is shown in the previous figure, is as follows. This distance was also used in the application of the Fourier transform, the results of which are presented in Section 3.4.5:(9)$d=\sqrt{{\left(X-x_{center}\right)}^{2}+{\left(Y-y_{center}\right)}^{2}}$

For each point, which is specific by X and Y coordinates, the absolute distance from the center of the concentric circles, which has *x_center_* and *y_center_* coordinates, was calculated.

The Piecewise Cubic Hermite Interpolating Polynomial (PCHIP) interpolation method was fitted to the resulting averaged trajectory [30,31].

This method returns a vector with interpolated values according to the shape-preserving piecewise cubic interpolation of x and y. The piecewise polynomial *f*(*x*) uses Formula (10).
(10)$$\left(x\right)=a{\left(x-x_{1}\right)}^{3}+b{\left(x-x_{1}\right)}^{2}+c\left(x-x_{1}\right)+d$$

**Figure 15 sensors-23-05872-f015:**
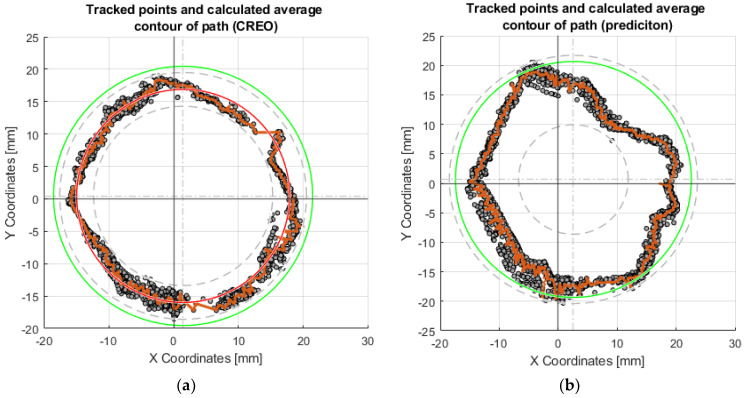
Graphs of the motion of the effector axis along the R20 circle: (**a**) Motion of the effector axis according to the values obtained from the CREO program; (**b**) Motion of the effector axis according to the values obtained from the prediction, trained on the motor control values from CREO.

The coefficients of the polynomial, in this case, are denoted by *a*, *b*, *c*, *d*. The intervals of the polynomial are denoted by *x* and *x*_1_. Compared to the linear interpolation method, this requires more computation time and is memory intensive. It requires at least four points. MATLAB was used to preprocess the collected data. This interpolated trajectory was used for calibration purposes, the shape of which is shown in Figure 15a.

The collected data (coordinates) from thirty repetitions of the effector axis movements on the surface were plotted in Figure 15. The effector axis performed a motion on a circle with radius R20. Figure 15a shows the coordinates of the points that were created by driving the device with the motor rotation values obtained from the CREO program (Section 3.2.1). It can be seen in the graph that the required circle R20 was not touched by the axis of the effector even once during the thirty repetitions. Training the neural network on the motor rotation values from CREO (Figure 15b) achieved better results in repeatability and touching the effector axis of the desired circle. The shape of the circle, created by moving the effector axis of the device in a post-comparison of the two graphs, achieved better results when controlled by the inverse kinematic values from CREO.

Each set of values in the tables obtained serves the purpose of comparing several ways of motor-control methods—control by values obtained from CREO, control by prediction values without calibration, calibration according to the mean circle, and calibration according to the average point curve. An important parameter is the deviation of the desired motion from the actual motion generated. These values are also expressed graphically as charts. For circular motion, the desired motion is represented by the green circle. By performing thirty repetitions of movement along the circle, the orange curve, which represents the average real position for the desired ones, allows for the evaluation of repeatability concerning the desired motions.

To achieve more accurate results, calibration of the servomotor position prediction model was proposed. The training values of the rotation of the motors according to the points formed on the central circle is shown in red in Figure 15a.

**Figure 16 sensors-23-05872-f016:**
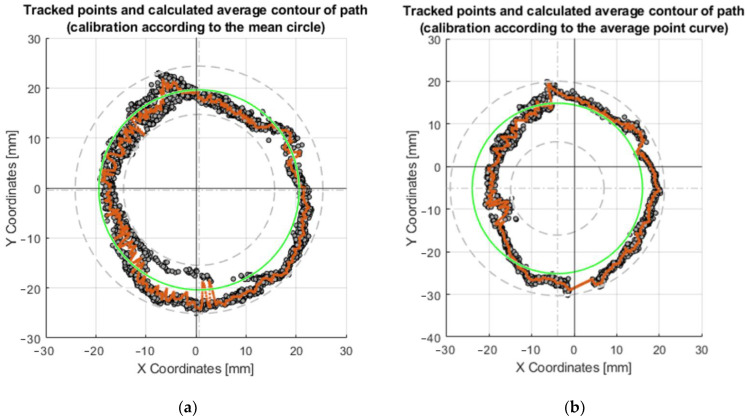
Graphs of the motion of the effector axis along the circle R20: (**a**) Movement of the effector axis according to the values obtained from the prediction, trained on the values of the mea circle; (**b**) Movement of the effector axis according to the values obtained from the prediction, trained on the values of the mean point curve.

The exact coordinates obtained by moving the axis of the effector along the circle R20 (radius 20 mm), are shown in Table A1. The deviations of the actual motion from the desired motion are important data to compare how the motors are controlled. In this case, it was the motion where the axis of the effector was to form a circle at the intersection with a 40 mm diameter millimeter paper (radius 20 mm). Since this was a circle formation, the most suitable way of movement was achieved by the difference between the inner radius and the outer radius. The formula could be written as x = inner radius + ((outer radius − inner radius)/2). The best results were obtained after calibration according to the mean circle (Figure 16a), where the result of the prediction was x = 19.92 mm (R19.92). This is the radius dimension that was closest to the desired radius (20 mm). Calibration according to the average point curve is shown on the Figure 16b.

**Figure 17 sensors-23-05872-f017:**
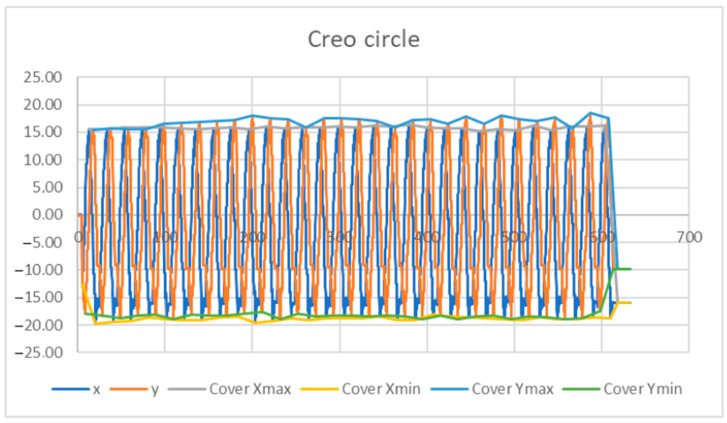
Envelope curve of maximum and minimum values.

**Figure 18 sensors-23-05872-f018:**
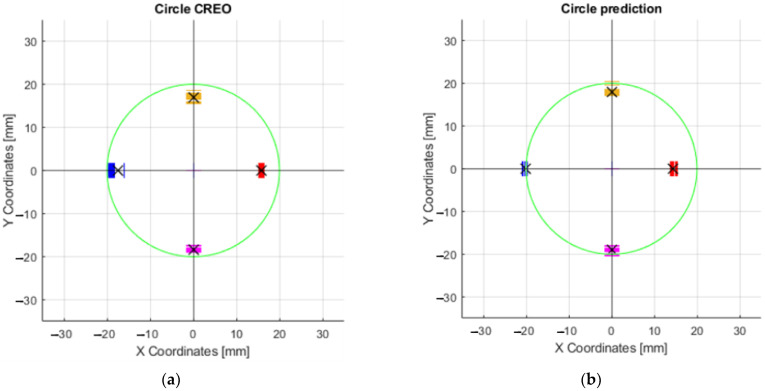
Graphs of the maximum and minimum values in the X and Y axes: (**a**) Movement of the effector axis according to the values obtained from the CREO; (**b**) Movement of the effector axis according to the values obtained from the prediction, trained on the motor control values from CREO.

**Figure 19 sensors-23-05872-f019:**
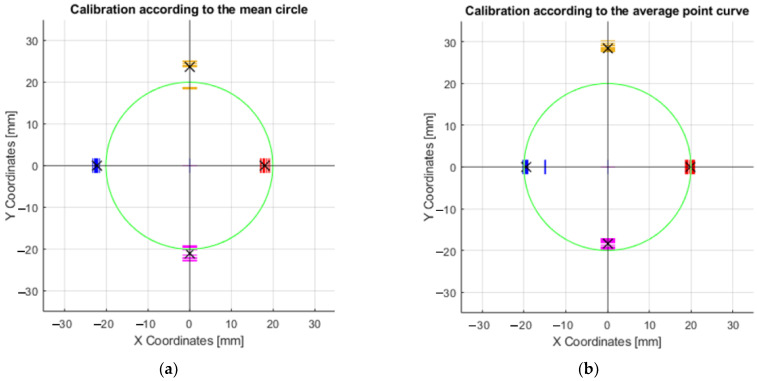
Graphs of maximum and minimum values in the X and Y axes: (**a**) Movement of the effector axis according to the values obtained from the prediction, trained on the values of the mean curve; (**b**) Movement of the effector axis according to the values obtained from the prediction, trained on the values of the curve.

From the chart shown in Figure 17, the maximum and minimum values that indicate the curves—Envelope Xmax, Envelope Xmin, Envelope Ymax, and EnvelopeYmin—were selected. These values were processed into graphs (Figure 18 and Figure 19). Envelopes, as a preliminary analysis tool, were created by connecting the local extrema using the “aggregate” function in MS Excel.

The graph that resulted from the above values during the movement of the axis of the effector along the circle of radius R20 is shown in Figure 18a. The same description of the circle was applied to the prediction for motor control (Figure 18b). By comparing these two graphs, it can be determined that the effector axis at the X − 20 Y0, X0 Y20, and X0 Y − 20 positions achieved more accurate positioning relative to the desired circle when the prediction used for control was applied. The average values shown by the black “X” symbol in Figure 18a are as follows: Y16.64, Y − 18.5, X15.8 and X − 17.47. The values in Figure 18b are Y17.87, Y − 18.88, X14.29, and X − 20.65.

After applying the center circle calibration, the plot shown in Figure 19a was produced. The calibration values produced by training the neural network on the values of the waveform are shown in the graph in Figure 19b. By comparing these graphs, it was found that the most accurate values of the positioning of the effector axis were found in the X-axis, after using the prediction produced by training the prediction on the curve values. By comparing the plots produced, it can be determined that the most accurate positions in the X and Y axes were achieved when steering after calibration according to the average point curve. These were the best results in the +X, −X, −Y axes. The average values shown by the black “X” symbol in Figure 19a are as follows: Y23.67, Y − 20.98, X17.98 and X − 22.22. The values in Figure 19b are Y28.5, Y − 18.27, X19.85, and X − 19.49.

#### 3.4.2. Processing the Results by Moving along the Circle R10

**Figure 20 sensors-23-05872-f020:**
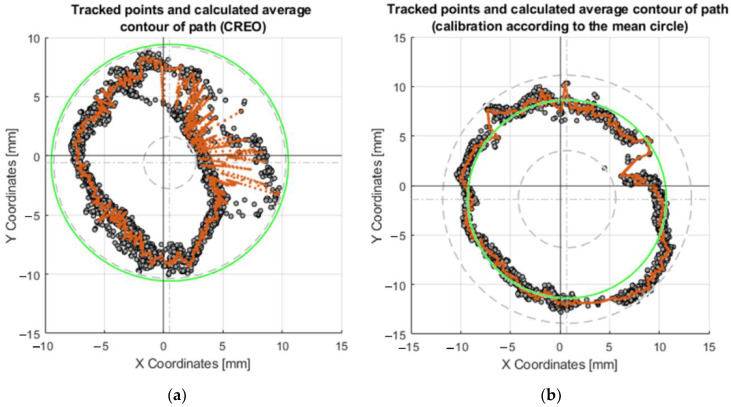
Plots of the motion of the effector axis along the R10 circle: (**a**) Motion of the effector axis according to the values obtained from the CREO; (**b**) Motion of the effector axis according to the values obtained from the prediction trained on the values of the mean circle.

To compare the control of the motors through inverse kinematics (Section 3.2.1) and calibration on the center circle, a motion on the R10 circle was created. It can be seen in the plots that more accurate values were achieved by the motion created by the prediction control (plot in Figure 20b) compared to the control via the values from CREO Figure 20a. It also achieved better repeatability results.

The exact coordinates obtained by moving the axis of the effector along the circle R10, are shown in Table A2. In this case, it is the motion in which the effector’s axis forms a circle at the intersection with a 20 mm diameter millimeter paper (radius R10). The equation for determining the central circle between the outer and inner is x = inner radius + ((outer radius − inner radius)/2). The best results were obtained following calibration using the mean circle, where the previous year’s result was x = 10.53 mm (R10.53). This is the closest radius dimension to the intended radius (R10).

#### 3.4.3. Processing the Results by Moving on the Line X30

**Figure 21 sensors-23-05872-f021:**
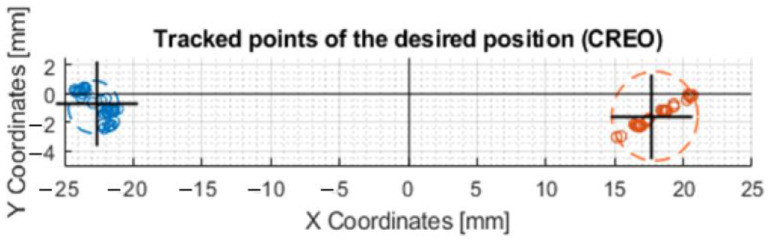

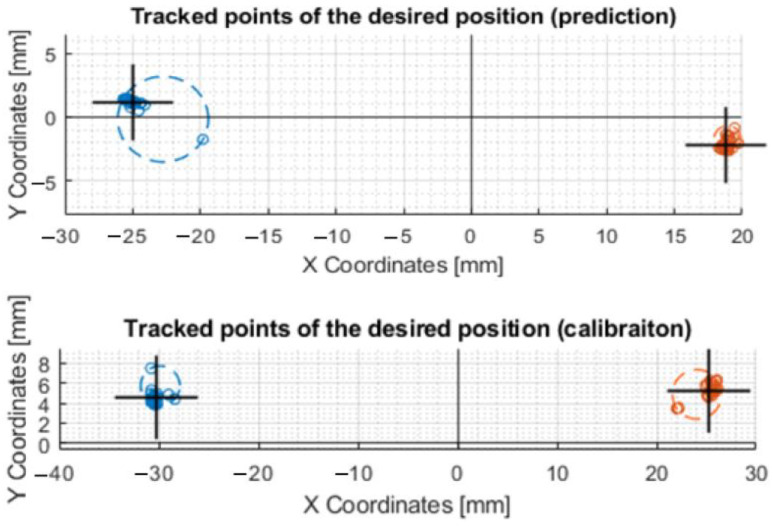
Movement of the axis of the effector along a straight line at coordinates X − 30 and X30. This figure shows a post-comparison of the mechanism control according to the values from CREO, prediction, and after calibration.

Another comparison of the motor control was made by moving along a line at X − 30,Y0 and X30,Y0 coordinates. In the plots that are shown in Figure 21, it can be seen that the X − 30 and X30 values were best achieved by the device when it was controlled by prediction after calibration.

The exact coordinates obtained by moving the effector axis along the line are shown in Table A3. That it was the most accurate movement in the X-axis when controlled by prediction after calibration is shown by the values written in the last row of the table: X − 30.3119 and X24.0485.

#### 3.4.4. Processing the Results by Moving on the Y50 Line

**Figure 22 sensors-23-05872-f022:**
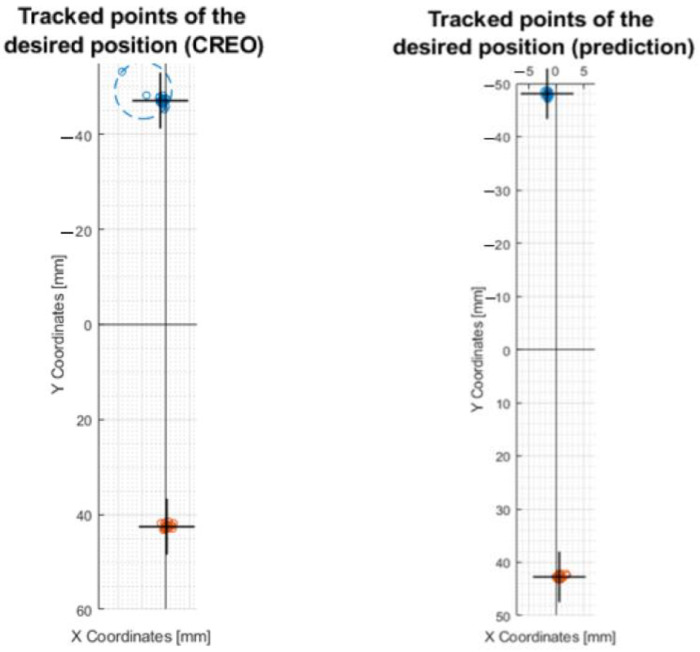
Movement of the axis of the effector along a straight line at Y − 50 and Y50 coordinates. This figure shows a comparison of the control of the mechanism according to the values from CREO and prediction.

A comparison of the mechanism control was also made on the movement of the effector axis at X0,Y50 and X0,Y − 50 coordinates. The processed motions on the graph can be seen in Figure 22, where control through inverse kinematics and control through prediction occurred.

The real values of the movement between points X0,Y50 and X0,Y − 50 are shown in Table A4. By comparing the two motor controls (Figure 22) it can be evaluated that the pros-mediated prediction control achieved better results: X − 1.6494, Y − 47.9879, and X0.5451, Y42.8011, respectively.

#### 3.4.5. Processing the Results by Moving on the Line X10Y30

**Figure 23 sensors-23-05872-f023:**
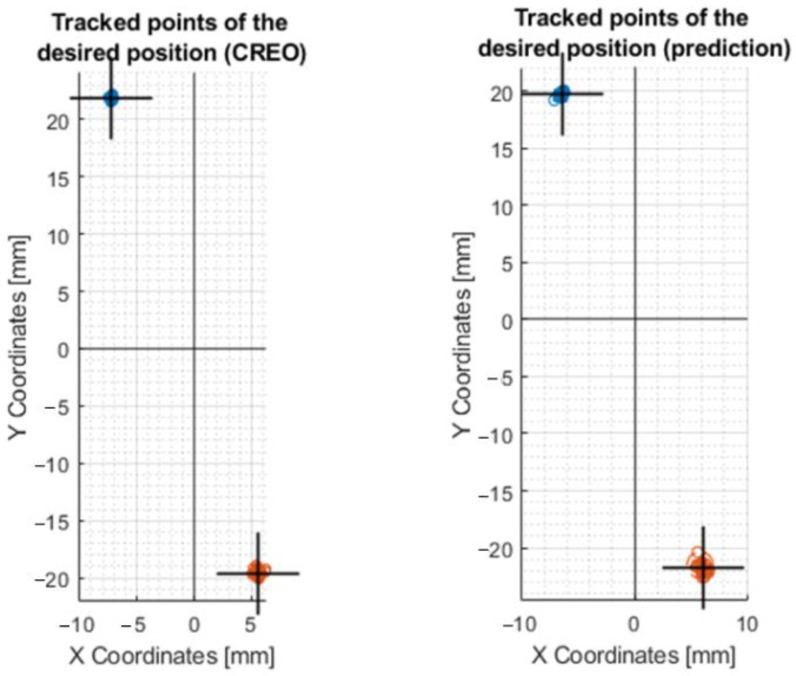
Movement of the axis of the effector along a straight line at coordinates X − 10Y30 and X10Y − 30. This figure shows the comparison of mechanism control by values from CREO and prediction.

A final comparison of the motor control was made at coordinates X − 10,Y30 and X10,Y − 30. Plots of the motion of the effector axis are shown in Figure 23.

According to the values shown in Table A5, it can be concluded that a more accurate movement on the X and Y axis was achieved by the device when it was controlled by the values from the prediction: X − 6.4536 Y − 19.7469 and X6.11 Y − 21.4966, respectively.

Given the generated motions, it can be determined that it is feasible to effectively use a neural network to control a mechatronic device with a parallel kinematic structure. The device when controlled through the predicted values achieves similar positioning performance as when controlled according to the values obtained from the inverse kinematics. Through the process of calibration, we successfully attained higher accuracy in the movements of the optical axis of the effector, aligning it more closely with the desired trajectories.

#### 3.4.6. Fourier Transform

An infinite sum of sines and cosines is used to represent the expansion of a periodic function *f*(*x*) into a Fourier series. The orthogonality relationships between the sine and cosine functions are used in the Fourier series [32].

Harmonic analysis is the computation and study of the Fourier series. An arbitrary periodic function can be divided into a collection of manageable terms that can be fed in, solved separately, and then combined to get the answer to the original puzzle or an accurate approximation of it [33].

**Figure 24 sensors-23-05872-f024:**
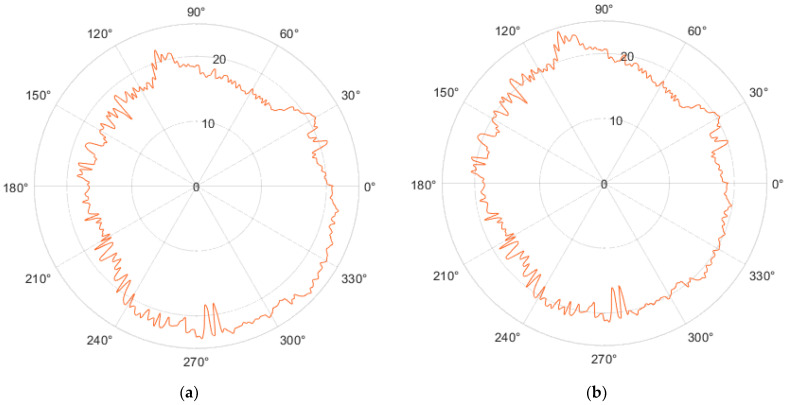
Using the Fourier transform to center the profile: (**a**) Movement of the real motion of the effector axis along the circle; (**b**) Moving the center of the profile to the X0 Y0 coordinates.

By removing the first harmonic component in the Fourier order, we achieved a shift (centering) of the center of the circle shown in Figure 24a, to the value X0,Y0—shown in Figure 24b.

It is possible to examine, for instance, the impact of technological production conditions on the geometric accuracy of the generated regions or to pinpoint the root causes of geometric errors (deviations) based on harmonic analysis [33].

**Figure 25 sensors-23-05872-f025:**
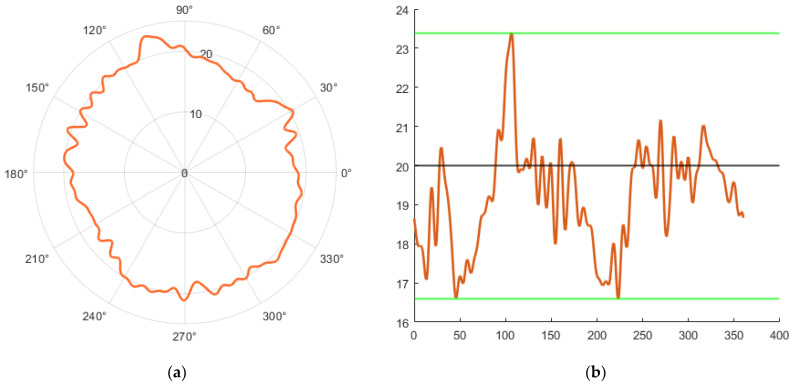
Profile of the circle after leaving 50 harmonic components: (**a**) Polar view; (**b**) Linear view.

By removing the first and leaving the remaining 50 harmonic components, the circularity profile in the polar view shown in Figure 25a was achieved. The linear view of this profile is shown in Figure 25b.

In pursuing this approach, our objective was to mitigate the potentially detrimental effects of vibrations that might have occurred during the measurement of effector movements. Additionally, we aimed to address inaccuracies that emerged from the detection of motion axes by the Tracker software (Section 3.3.1).

## 4. Discussions

In this work, we found that the created mechatronic device can be controlled by neural networks. The proposed calibration method demonstrates its efficacy in facilitating the attainment of more precise values, whereby the training process allows the alignment of the actual movement of the optical axis with the desired position. If we compare, for example, the motion along the R10 circle, when the motors were controlled by prediction they achieved much more accurate movements concerning the desired ones, in contrast to the control of the motors utilizing inverse kinematics (Section 3.4.2).

Using the Fourier transform, we achieved the centering of the resulting circle on the X0 and Y0 coordinates of the graph. The contribution of this work can be negotiated in several ways. Education—students will be exposed to a real device through a fabricated device in which artificial intelligence models—prediction, machine vision, and voice recognition—are implemented. Research—the constructed device can be used for several demonstration tasks in the field of exploring AI algorithms.

Considering other established works dealing with prediction in the field of motors, this paper describes the simultaneous control of 3 motors in order to reach a specific effector point, according to previously detected motions. From this point of view, it is a unique approach to dealing with motor control, in which other AI elements can be implemented in further research. Among the main advantages of this research can be included the speed of control generation for virtually any kinematic structure. The disadvantage lies in the inaccuracy of the resulting device motions.

In the design process, a deliberate constraint was imposed to use exclusively the production technologies and materials currently available at the laboratory. Reasonable clearances in the mechanism were necessary to achieve the desired functions. The control system was limited as only the values from the inverse kinematics and predicted values were used to control the motors. Even though a model of the control structure of the designed mechanism was created in Section 3.2.2 using Matlab, this control was not included in the experimental part of this paper. The depicted control structure can help readers to create their own “Agile eye” with control via Matlab. To make the replication of experiments easier, only open-source libraries were used.

The time between running the program on the RPi and moving the constructed device (response time) is 1.62 s. According to the technical specifications of the Waveshare MG90S, this servo motor has a resolution of 10 bits, which means it can have up to 1024 different positions. The motors used in the device are characterized by “high resolution”. Repeatability for movement along the R20 circle was best when the device was controlled by precalculated values from inverse kinematics (5.22 mm). However, when moving along the R10 circle, it reached more accurate values when controlled via prediction (6.61 mm). The achieved repeatability values, despite being quite high, thus demonstrated the possibility of using neural networks in applications where it is not necessary to achieve precise movements—tracking objects by camera systems over a large area, sensing products on a conveyor, etc.

## 5. Conclusions

According to the measured and processed values, it is possible to say that the device can be controlled by neural networks (prediction). By calibration, it is possible to achieve more accurate results, or in other terms results closer to the desired ones. The inaccuracies and randomness of positioning that occurred in the circular and linear movements were caused mainly by the loose fit of joint pins of the Agile Eye mechatronic device, and the backlash of the servomotors. More precise parts made of stiffer material would result in overall higher accuracy and precision. Using such construction for replication of the control process described in this paper would achieve results with higher precision. However, higher precision would make measuring and evaluating of impacts of changes in the controlling process harder. The described device is meant mainly for experiments regarding various methods of control. Therefore, some errors in the mechanism are even desirable.

In this paper, we did not try to prove that this method of motor control is the best, but to test whether it is possible to control motors in a complex parallel kinematic structure utilizing neural networks. Even though we avoided the real use of the generated rotation matrices in the program, we were able to achieve the desired motions with the effector. The proposed calibration also produced excellent results. Several movement patterns were created and each was repeated 30 times. These movements were—Movement on a circle with radius R20/R10, Movement on line X30/Y50 and X10Y30. The results uniquely demonstrated the ability to execute the default motions, according to the predicted values. The research demonstrated that the generated backlash in the created device can be eliminated to some extent through calibration. The paper can serve as a basis for further research in the field of motor control and applications of AI algorithms.

Further work on the project involves using machine vision for person detection and voice command recognition—the Arducam module located in the effector has a camera and microphone implemented for these purposes.

## References

## Data Availability

The data presented in this study are available on request from the corresponding author.

## Appendix A

**Table A1 sensors-23-05872-t0A1:** The exact values of the achieved coordinates from the plots in Figure 15 and Figure 16.

Circle R20	*x_center_* [mm]	*y_center_* [mm]	Dimension of the Annular Area [mm]	Radius Inner [mm]	Radius Outer [mm]
CREO	1.4529	0.45085	5.221	13.827	19.0481
Prediction	2.584	0.64836	11.7513	9.2923	21.0436
Calibration according to the mean circle	0.61807	−0.38082	9.6603	15.0923	24.7526
Calibration according to the average point curve	−3.9477	−5.135	14.2048	10.9642	25.169

**Table A2 sensors-23-05872-t0A2:** Exact values of the coordinates obtained from the plots in Figure 20.

Circle R10	*x_center_* [mm]	*y_center_* [mm]	Dimension of the Annular Area [mm]	Radius Inner [mm]	Radius Outer [mm]
CREO	0.52421	−0.57734	7.5812	2.2057	9.7869
Prediction	−0.45999	0.19545	6.6117	3.3813	9.9929
Calibration according to the mean circle	0.71054	−1.3825	7.623	4.9045	12.5276

**Table A3 sensors-23-05872-t0A3:** The exact values of the achieved coordinates from the plots in Figure 21.

Line X30	Mean A [X;Y]	Circle A [X;Y]	Circle Radius [A;B]	Mean B [X;Y]	Circle B [X;Y]
CREO	−22.6290; −0.7716	−22.8759; −0.9357	1.8368; 3.1305	17.7390; −1.6877	17.9179; −1.5837
Prediction	−24.9526; 1.1181	−22.7572; 0.1707	3.3431; 0.9870	18.8197; −2.1449	18.9206; −1.6785
Calibration according to the mean circle	−30.3119; 4.6014	−29.8509; 5.7452	1.96; 2.4627	25.2273; 5.2472	24.0485; 4.8840

**Table A4 sensors-23-05872-t0A4:** The exact values of the achieved coordinates from the plots in Figure 22.

Line Y50	Mean A [X;Y]	Circle A [X;Y]	Circle Radius [A;B]	Mean B [X;Y]	Circle B [X;Y]
CREO	−47.2469; −1.0282	−49.2846; −4.6818	5.9688; 1.4017	42.4793; 0.1806	42.1856; 0.3747
Prediction	−47.9879; −1.6494	−47.9684; −1.5761	0.8949; 1.0737	42.8011; 0.5451	42.5573; 0.9177

**Table A5 sensors-23-05872-t0A5:** The exact coordinate values obtained from the plots in Figure 23.

Line X10Y30	Mean A [X;Y]	Circle A [X;Y]	Circle Radius [A;B]	Mean B [X;Y]	Circle B [X;Y]
CREO	−7.1995; 21.8294	−7.1865; 21.8432	0.3023; 0.6011	5.5410; −19.5651	5.6359; −19.4667
Prediction	−6.4536; 19.7469	−6.6761; 19.5895	0.6207; 1.1513	6.11; −21.8049	5.8308; −21.4966
